# The Effect of Availability of Manpower on Trauma Resuscitation Times in a Tertiary Academic Hospital

**DOI:** 10.1371/journal.pone.0154595

**Published:** 2016-05-02

**Authors:** Timothy Xin Zhong Tan, Nathaniel Xin Ern Quek, Zhi Xiong Koh, Nivedita Nadkarni, Kanageswari Singaram, Andrew Fu Wah Ho, Marcus Eng Hock Ong, Ting Hway Wong

**Affiliations:** 1 Yong Loo Lin School of Medicine, National University of Singapore, Singapore, Singapore; 2 Department of Emergency Medicine, Singapore General Hospital, Singapore, Singapore; 3 Centre for Quantitative Medicine, Duke-NUS Graduate Medical School, Singapore, Singapore; 4 Trauma Service, Department of General Surgery, Singapore General Hospital, Singapore, Singapore; 5 Health Services and Systems Research, Duke-NUS Graduate Medical School, Singapore, Singapore; University of South Florida, UNITED STATES

## Abstract

**Background:**

For trauma patients, delays to assessment, resuscitation, and definitive care affect outcomes. We studied the effects of resuscitation area occupancy and trauma team size on trauma team resuscitation speed in an observational study at a tertiary academic institution in Singapore.

**Methods:**

From January 2014 to January 2015, resuscitation videos of trauma team activated patients with an Injury Severity Score of 9 or more were extracted for review within 14 days by independent reviewers. Exclusion criteria were patients dead on arrival, inter-hospital transfers, and up-triaged patients. Data captured included manpower availability (trauma team size and resuscitation area occupancy), assessment (airway, breathing, circulation, logroll), interventions (vascular access, imaging), and process-of-care time intervals (time to assessment/intervention/adjuncts, time to imaging, and total time in the emergency department). Clinical data were obtained by chart review and from the trauma registry.

**Results:**

Videos of 70 patients were reviewed over a 13-month period. The median time spent in the emergency department was 154.9 minutes (IQR 130.7–207.5) and the median resuscitation team size was 7, with larger team sizes correlating with faster process-of-care time intervals: time to airway assessment (p = 0.08) and time to disposition (p = 0.04). The mean resuscitation area occupancy rate (RAOR) was 1.89±2.49, and the RAOR was positively correlated with time spent in the emergency department (p = 0.009).

**Conclusion:**

Our results suggest that adequate staffing for trauma teams and resuscitation room occupancy are correlated with faster trauma resuscitation and reduced time spent in the emergency department.

## Introduction

Improvements in hospital trauma systems can lead to better outcomes and survival [1]. Trauma teams reduce the time taken for resuscitation and total time spent in the emergency department (ED) [2] [3].

One approach to improving trauma resuscitation outcomes and standards is the auditing of team dynamics [4] [5] as well as other aspects of the hospital trauma system [6]. For example, Alavi-Moghaddam utilized computer modelling and queuing theory analysis to unmask bottlenecks in laboratory and ED consultant capacity [7]. Once these were circumvented, significant improvements in patient flow were observed. A larger study by the Scottish Trauma Audit Group demonstrated significant improvements in trauma patient survival (from 65% to 79%) post-implementation of a trauma service audit program [8]. In our study, we sought to identify barriers to time-efficient resuscitation in a tertiary academic medical center in Singapore equipped with round-the-clock emergency medicine specialist coverage and stay-in general surgery and orthopedic teams (at least senior resident level) [9], with the goal of subsequent improvement to trauma workflow.

## Methods

The use of video recording in trauma as a training technique has been previously described in literature [10]. We used video recordings to identify areas for improvement in our resuscitation procedures. A high-resolution motion-activated closed circuit television system (CCTV) had been installed in the resuscitation area of the ED for the audit of cardiopulmonary resuscitation [11]. For privacy protection, approval for access to video footage is granted only for authorized personnel involved in quality improvement programs. From January 2014 to January 2015, videos of all trauma team activations were extracted for analysis with the exception of cases with an Injury Severity Score (ISS) of <9 and patients dead on arrival (no spontaneous cardiac output or respiratory effort at the time of arrival). Inter-hospital transfers and up-triaged patients were also excluded because initial resuscitation happened outside the CCTV-monitored resuscitation area. The criteria used for trauma team activation in our hospital include the minimum criteria in the American College of Surgeons Guidelines [12] [13] [14].

### Definitions

Resuscitation Area Occupancy Rates at t = 0 (RAOR) were extracted from the CCTV footage of all 6 bays at the start of resuscitation. An RAOR of 6 refers to a full resuscitation area with all 6 bays filled, while an RAOR of 1 refers to a resuscitation area with only 1 out of the 6 bays filled (i.e. only the trauma patient in question).

The onset of resuscitation (t = 0) was defined as the time at which the patient’s trolley is parked in the resuscitation area, allowing interventions to begin.

The end of resuscitation was defined as the time when the patient was wheeled off for final disposition out of the ED.

As trauma team size is known to fluctuate in the course of a resuscitation, the trauma team size utilized for this study was defined as the largest size of the team with the trauma team leader present at bedside from the start of resuscitation until eventual disposition out of the ED. Physicians, nurses, and medical students were included only if they actively assisted in resuscitation.

### Video Analysis

Video footage was viewed independently by at least one of the trained co-investigators of the study, with quantitative timings and data distilled for subsequent analysis. Standardization of time-point capture and definitions was performed for the first ten videos with at least two co-investigators viewing the videos independently and recording timings for subsequent inter-rater comparisons and standardization of methods. The observers did not review any videos where they were themselves part of the trauma team activation.

Data extracted included: resuscitation manpower characteristics (trauma team size, trauma team leader arrival time, ED consultant arrival time, and RAOR), interventions (airway assessment, timing of intubation attempts, use of supplementary oxygen, timing of vascular access, timing and type of immobilization, timing of Focused Assessment with Sonography for Trauma (FAST), and timing of logrolls), and process-of-care time intervals (time to assessment/intervention/adjuncts, resuscitation procedures, imaging, and eventual disposition). Interventions were identified and classified by the Advanced Trauma Life Support (ATLS) principles of Airway, Breathing, Circulation, Disability, and Exposure.

### Other data sources

Additional demographic and clinical data obtained from the hospital trauma registry and chart review included: patient demographics (age, gender), injury mechanism (blunt vs. penetrating injury, intentional vs. unintentional), ISS, Revised Trauma Score (RTS), and eventual patient outcomes (Total Length of Stay (LOS), and in-hospital survival).

### Ethical Approval

Ethical approval for this study was granted by the Singapore General Hospital Institutional Review Board. All data was stored on password-protected computers within a locked room (the trauma office) and anonymized prior to statistical analysis for privacy protection. Due to the retrospective observational nature of the study, the study was granted exemption from requiring patient consent by the institutional review board.

## Results

### Demographics of Study Cohort

Out of a total of 185 trauma activations within this 13-month period, 96 met inclusion criteria. Sixty-six patients had an ISS<9, 4 were dead on arrival, and there were 19 patients who were initially under-triaged or transferred from other institutions. Eighteen video-captures were missed due to data overwriting within the system, and 8 videos were corrupted. This is however unlikely to introduce significant bias as the missing and corrupted video files did not occur at any particular time of day or day of week. Videos of 70 patients were analyzed (Table 1).

**Table 1:** Population and Injury Demographics.

Characteristic	N (%) / Mean (SD) / Median (interquartile range)
Trauma Activations with Videos Analyzed	70
Age of Patients in Years	41.7 (17.9)
Gender	
- Male (%)	51 (72.9)
Injury Type	
- Blunt (%)	66 (94.3)
Mechanism of Injury	
- Road Traffic Injury (%)	44 (62.9)
- Fall From height (%)	15 (21.4)
- Assault (%)	7 (10.0)
- Industrial Accident (%)	1 (1.4)
- Unknown (%)	3 (4.3)
Injury Severity Score	14.00 (9.0–22.0)
Revised Trauma Score	7.841 (6.904–7.841)
Probability of Survival	98.30 (93.40–99.60)
ICU Length of Stay in Days	0.00 (0.00–0.625)
Total Length of Stay in Days	4.75 (1.75–18.0)
Discharge Status	
- Home (%)	54 (77.1)
- Step-down Care (%)	9 (12.9)
- ED Death (%)	2 (2.9)
- Ward Death (%)	5 (7.1)

Of the patients whose videos were captured for analysis, 51 (72.9%) were male, ages ranging from 17 through 77, with a mean age of 41.7 years. Most were blunt injuries (n = 66, 94.3%). Road traffic injuries (RTI) were the most common mechanism of injury (n = 44, 62.9%), followed by falls from height (n = 15, 21.4%), assault (n = 7, 10.0%), and industrial accidents (n = 1, 1.4%). The median ISS was 14.00 (interquartile range [IQR] 2.0–22.0) and the median RTS was 7.841 (IQR 6.904–7.841). The median LOS in hospital was 4.75 days (IQR 1.75–18.0). 77.1% of our trauma patients were eventually discharged home and 12.9% were discharged to step-down care, including rehabilitation hospitals.

### Human Resources and Intervention Frequency

We analyzed the effects of the following resource availability variables on resuscitation timings: Trauma Team size, RAOR, and Frequency of interventions.

#### 1. Trauma team size

Team sizes ranged from 4 to 15, with a median team size of 7.00 (IQR 5.75–8.00), out of which 4.79 (SD = 1.55) were physicians.

Pearson’s correlation showed that team size was correlated with shorter time to airway assessment and eventual disposition (Table 2).

**Table 2:** Effect of Team Size Variation (<7 vs. ≥7) on Outcome and Time Interval Factors.

Time Intervals	Median (minutes)	IQR (minutes)	Pearson Correlation (r-value)	P-value	95% CI
Time from start to:					
- Arrival of senior ED doctor	0.00	0.00–2.03	-0.053	NS	
- Arrival of Trauma Team Leader	11.95	8.48–20.45	0.045	NS	
- Monitors attached	3.38	1.51–7.62	-0.148	NS	
- **Airway assessment**	**0.38**	**0.00–1.33**	**-0.313**	**0.008**	**-0.510, -0.085**
- IV plug attempt	2.20	0.67–5.12	-0.186	NS	
- 1st blood sample	4.93	2.37–7.83	-0.118	NS	
- Patent IV plug	5.52	2.88–8.75	-0.190	NS	
- Start of Exposure	1.12	0.28–3.43	-0.138	NS	
- Completion of Exposure	7.05	2.28–12.03	-0.229	0.076	-0.440, 0.006
- 1st log roll	19.86	9.78–27.28	-0.036	NS	
- 1st FAST	7.66	3.87–14.92	-0.060	NS	
- 1st X-ray	32.83	24.54–46.35	-0.099	NS	
- Leaving for 1st CT	46.58	35.43–70.13	-0.250	0.058	-0.541, -0.016
- Final intervention	143.98	110.33–183.83	-0.069	NS	
- **Disposition**	**184.42**	**142.36–241.62**	**-0.247**	**0.039**	**-0.455, -0.013**

Using Spearman’s correlation, we compared team sizes smaller than the median team size of 7 to team sizes of 7 or more. This also showed longer ATLS primary survey process-of-care time intervals for smaller teams: Time to airway assessment (p = 0.045), Time to start of exposure (p = 0.006), and Time to completion of exposure (p = 0.034), as well as longer Time to 1^st^ Computed Tomography (CT) (p = 0.002).

#### 2. Resuscitation Area Occupancy Rate (RAOR)

RAORs at t = 0 were extracted from the CCTV footage. RAORs were not extracted for nine cases due to human error. Repeat measurements of the RAOR were performed over the first 5 minutes for the initial 10 cases, and it was found that the RAOR did not change for most cases. There was a significant positive correlation between RAOR and time to eventual disposition (Pearson coefficient 0.330, p = 0.009).

#### 3. Frequency of interventions

Potential impediments to assessment, in particular, type of immobilization (C-collar, splint/traction device, spinal board, and scoop), and the need for repeat interventions (FAST, repeat intubation or repeat vascular access attempts) were also analyzed. These did not affect the process-to-time outcome measures, although repeat FAST was more common in more severely injured patients.

### Process-of-care Time Intervals

**Fig 1:**
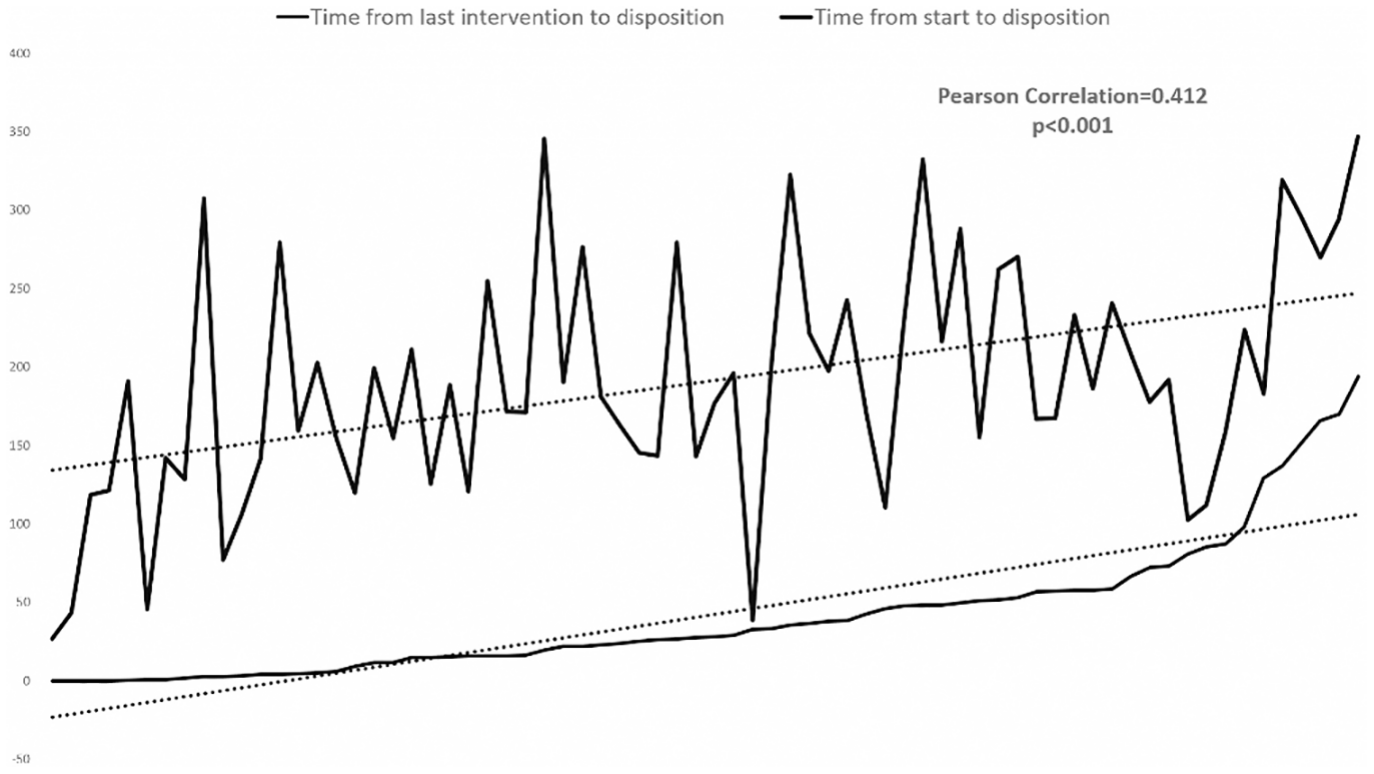
Time from last intervention to disposition versus Time from start to disposition.

In this study, time intervals were mostly viewed as outcomes. Airway assessment coinciding with arrival of the patient in the bay occurred 31.4% of the time (n = 22). Overall, a median time from arrival to airway assessment of 23.00 seconds was observed. Notably, “time between last intervention and disposal” was also found to be the greatest contributor to the total time a patient spent in the ED (0.412, p <0.001) (Fig 1).

### Limitations

Limitations of our study include the small study sample of 70. Other limitations include the variation of team size over the course of resuscitation. Ideally, every team member should be tagged, their time spent in the resuscitation area tallied up, and the average team size-per-hour calculated. This was deemed unfeasible, and we instead used the team size at the arrival-of-trauma-team-leader as a surrogate for overall team size. While largely accurate, there were times when the team leader arrived only after the majority of procedures had been completed. This parameter may therefore not be fully representative of the actual staffing during the acute early phases of the resuscitation.

Furthermore, audio input of the proceedings was unavailable due to the nature of the equipment and the lack of microphones in situ, which would have provided us an insight into the quality of team interactions, rather than just the availability of manpower for the trauma activation. Future studies planned at our institution will analyze the role of teamwork and inter-professional interactions [15] in trauma resuscitation efficacy.

Lastly, there were several other variables unaccounted for in this study of a highly dynamic and multifactorial environment. These include team familiarity, nurse and allied healthcare staff expertise and seniority, and a differentiation of patients by the number of specialties involved.

Despite these limitations, our results reveal interesting data on manpower availability and resuscitation timings, serving as a baseline from which we are planning future interventions to speed up resuscitation.

## Discussion

In an ideal world, the ATLS approach to trauma resuscitation would be a coordinated, seamless process that flows from one step to the next, with simultaneous interventions where possible [3]. The reality of trauma resuscitation is, however, a complex, multifactorial, and multidisciplinary collaboration where team performance and eventual outcomes are highly variable [16].

This study has demonstrated that manpower availability beyond what is conventionally described as the “optimum team size” can result in shorter timings of trauma resuscitation. Firstly, a larger trauma team size results in quicker completion of the primary survey, including airway assessment, initiation, and completion of exposure. Forty-one (58.6%) of our resuscitations were conducted with a team size of seven or more. Other time intervals, although recorded, did not yield statistically significant results as the intervals in question were larger and therefore more susceptible to external variability/influences. Ideally airway is the first step in resuscitation. In our video analyses however, we realized that this did not always happen immediately on patient arrival, although it was the case in the majority of the videos. Correlating patient outcomes with resuscitation timings did not show any statistical significance, partly due to the small numbers, and also because slower resuscitation timings were observed for patients with better RTS and ISS scores where there may have been a reduced sense of urgency.

Some authors suggest the presence of an ‘optimum’ number beyond which productivity appears to decrease [17] [18] [19]. Reasons proffered include increased communication barriers, role and goal ambiguity, and physical crowding of the resuscitation area. Our study suggests that larger team sizes enable more efficient conduct of the steps required in trauma resuscitation without a hindrance to teamwork that was previously suggested [16]. We also looked for, but did not detect a statistically significant correlation between team size and time of day. Although some might predict that larger team sizes are noted during normal office hours, we found this to be not the case, as we have sufficiently staffed stay-in general surgery and orthopedic teams. We also measured bedside arrival times of senior ED doctors (consultants and above) and trauma team leaders (General Surgery Registrar). However, this did not appear to have any correlation to process-of-care timing intervals.

The other resuscitation resource explored was RAOR. There was a positive correlation between RAOR and time from resuscitation start to eventual disposition, i.e. the more patients in the resuscitation bay, the slower the trauma resuscitation. Although the mean RAOR was 2.02, almost a quarter of cases (21.3%) experienced full area occupancy (RAOR of 6). We also occasionally noticed the early disposition of patients from the panels in order to free up space for newly arrived patients. These findings have implications for resource management in busy EDs.

Lastly, the time between last intervention and disposal, i.e. time in ED spent simply waiting for disposition, is significantly correlated with the total time spent in the ED. In other words, bed waiting time is a statistically significant source of delay, the reduction of which will theoretically lead to improvements in RAOR and overall trauma resuscitation timings. Interestingly, in our study, “time between last intervention and disposal” was the greatest contributor to total time spent in the ED. This suggests that “bed block”, or the lack of in-patient beds, even after efficient trauma resuscitation, can lead to increased ED waiting time [20]. While there are studies showing that time spent in ED is an independent predictor of morbidity and mortality [21], the underlying reasons are not clear and it is worth studying whether the cause of mortality is due to prolonged bed waiting time, slow resuscitation, or both.

In summary, manpower availability affects trauma resuscitation times, even in a well-staffed tertiary academic hospital with stay-in specialist availability. A larger trauma team size speeds up the completion of ATLS primary survey, and our study does not show any reduction in efficiency from larger team sizes. We also note the significance of resuscitation area occupancy on overall time spent in the ED, indicating a possible need for further resource management. Finally, time from final intervention to disposition is a significant contributor to total ED wait time, the reduction of which will likely lead to improvements in eventual patient outcomes.
